# Robustness of Distinctive Facial Features in Prader-Willi Syndrome: A Stereophotogrammetric Analysis and Association with Clinical and Biochemical Markers in Adult Individuals

**DOI:** 10.3390/biology11081148

**Published:** 2022-07-30

**Authors:** Claudia Dolci, Antonello E. Rigamonti, Annalisa Cappella, Daniele M. Gibelli, Graziano Grugni, Diana Caroli, Chiarella Sforza, Alessandro Sartorio

**Affiliations:** 1 Laboratory of Functional Anatomy of the Stomatognathic System (LAFAS), Department of Biomedical Sciences for Health, Università degli Studi di Milano, 20133 Milan, Italy; annalisa.cappella@unimi.it (A.C.); daniele.gibelli@unimi.it (D.M.G.); chiarella.sforza@unimi.it (C.S.); 2 Department of Clinical Sciences and Community Health, University of Milan, 20129 Milan, Italy; antonello.rigamonti@unimi.it; 3 UO Laboratory of Applied Human Morphology, IRCCS Policlinico San Donato, 20097 San Donato Milanese, Italy; 4 Division of Auxology and Metabolic Diseases, Istituto Auxologico Italiano, Istituto di Ricovero e Cura a Carattere Scientifico (IRCCS), 28824 Verbania, Italy; g.grugni@auxologico.it (G.G.); sartorio@auxologico.it (A.S.); 5 Experimental Laboratory for Auxo-Endocrinological Research, Istituto Auxologico Italiano, Istituto di Ricovero e Cura a Carattere Scientifico (IRCCS), 28824 Verbania, Italy; d.caroli@auxologico.it

**Keywords:** Prader-Willi syndrome, adult, facial features, anthropometry, stereophotogrammetry

## Abstract

**Simple Summary:**

Prader-Willi syndrome (PWS) is a rare genetically determined neurodevelopmental disorder usually associated with a peculiar facial appearance. There is poor information concerning the relationships between the facial dysmorphism in PWS and other manifestations of the disease and its treatment. This exploratory study aimed to investigate this aspect, since this knowledge might aid in both diagnosis and monitoring of the disease. Three-dimensional facial images of fifteen Caucasian adult individuals with PWS were acquired through stereophotogrammetry; linear distances and angles were then calculated and compared with those of groups of healthy subjects matched for sex and age, obtained using the same noninvasive procedure. Individuals with PWS were evaluated for endocrine/metabolic dysfunction and nocturnal respiratory function, and their associations with facial features were tested. Some facial characteristics did not show any associations with other manifestations of PWS; therefore, they could constitute robust distinctive facial features and contribute to the diagnosis of the disorder. Conversely, other facial characteristics showed relationships with clinical and biochemical markers of PWS, suggesting their potentially useful role in the clinical monitoring and management of the disease. Further studies are needed to confirm these findings.

**Abstract:**

Background: Prader-Willi syndrome (PWS) is a rare genomic imprinting disorder associated to a complex neurodevelopmental phenotype and a distinctive facial appearance. The study investigated the relationships between the quantitative facial dysmorphism in PWS and clinical and biochemical markers of the disease and its treatment. Methods: Facial images of 15 Caucasian adult individuals with PWS (8 males, 42 ± 5 years; 7 females, 37 ± 8 years; BMI 38.87 ± 8.92 kg/m^2^) were acquired through stereophotogrammetry. From the 3D coordinates of 38 landmarks, linear distances and angles were calculated; they were expressed as z-score values by referring to 403 healthy subjects matched for age and sex and compared by Student’s *t*-test with Bonferroni correction for multiple testing. Patients underwent auxological and biochemical assessment of endocrine/metabolic dysfunction and nocturnal respiratory function. An exploratory correlation analysis was performed to investigate their associations with the facial phenotype; uncorrected *p*-values were used. Results and Conclusions: Individuals with PWS showed decreased bifrontal diameter, facial depths, palpebral fissures, mandibular ramus length, lower vermillion height, and modified relative position of exocanthia and nasion. Since these characteristics did not show any associations with clinical and biochemical markers of PWS, they could constitute robust distinctive facial features and contribute to the diagnosis of the disorder. Individuals with PWS showed also a larger mandibular width with smaller gonial angles, thinner upper vermillion, greater inclination of the orbit relative to the Frankfurt plane, and a smaller angle of the auricles versus the facial midplane. Relationships between these facial anthropometric features and body composition, glucidic metabolism indexes, nocturnal hypoxemia episodes, or duration of GH treatment were found, suggesting their potentially useful role in the clinical monitoring and management of the disease. However, they need to be confirmed by subsequent dedicated studies.

## 1. Introduction

Prader-Willi syndrome (PWS) is a rare imprinting disorder caused by the lack of expression of genes in the paternally derived region q11–q13 on chromosome 15. Its prevalence is estimated between 1:10,000 and 1:30,000 individuals, affecting both sexes equally, with no propensity for specific ethnic groups [1,2]. The disease usually occurs sporadically, arising from three main mechanisms which identify distinct genetic subtypes. A paternal deletion (DEL) within the PWS critical region is the most common cause, being responsible for about 60–65% of cases, maternal uniparental disomy (UPD) occurs in 30–35% of cases, and imprinting defects turn off paternal genes on chromosome 15 in 1–4% of cases [3].

PWS manifests itself as a complex phenotype that significantly varies from birth to adulthood, with interindividual variability. During early childhood, PWS is characterized by severe hypotonia with feeding problems. Soon later, symptoms move toward hyperfagia, resulting in a gradual development of obesity and increasing risk of comorbidities, including type II diabetes mellitus and sleep breathing disorders. In addition to developmental delay with mild cognitive impairment and maladaptive behaviours, patients with PWS experience consequences related to hypothalamic dysfunction. Multiple endocrinopathies also occur, such as growth hormone (GH) deficiency, central hypogonadism, hypothyroidism, and central adrenal insufficiency [4,5].

Individuals suffering from PWS may also exhibit typical somatic traits, including short stature, small hands and feet, and high occurrence of scoliosis and kyphosis, together with a peculiar facial appearance [1,6]. According to consensus diagnostic criteria established by Holm et al. [7], characteristic facial features include narrow bifrontal diameter, almond-shaped palpebral fissures, and small appearing mouth with thin upper lip and down-turned corners; those features may not be clear from birth and evolve over time [8,9]. Furthermore, a dolichocephalic head shape is common in infancy. Among the major clinical diagnostic criteria for PWS [10], a typical facial phenotype was found to be the least sensitive in selecting patients with PWS, and currently it is not included among the clinical findings that should prompt diagnostic genetic testing [11]. Nevertheless, since at least a part of the overall phenotype of PWS overlaps with other disorders, an accurate examination of the face to detect specific features can support differential diagnosis.

Despite general aspects of facial dysmorphism associated with PWS having been known for a long time, they often refer to a qualitative morphological evaluation of the face, and subtle but distinctive anomalies could have been missed so far. Few studies on large numbers of subjects employed quantitative approaches to investigate linear and angular features of the face in PWS, being based on 2D photographs or radiographs, with unavoidable limits, including distortion of images, landmark identification, and reliability [12,13,14,15,16]. Direct anthropometry was also used, but only for few measurements of the craniofacial district [17]. Therefore, a detailed anthropometric description of PWS facies is not yet available. Three-dimensional (3D) optical devices have been increasingly used in the quantitative evaluation of facial dysmorphisms, both for research purposes and clinical practice. Particularly, stereophotogrammetry is gaining a leading role in clinical dysmorphology, due to its reliability, non-invasiveness, and simplicity of use [18,19].

Currently, there is poor information about the relationships between facial morphology in PWS and other clinical findings, including biochemical markers of endocrine/metabolic dysfunction and sleep breathing disorders. Indeed, specific dysmorphic facial signs could have a predictive value for more serious complications of the disease. For example, a recent study on individuals with Marfan syndrome highlighted a positive correlation between a group of oro-facial defects and systemic alterations of the disease including the aortic dilation, which predisposes to the life-threatening artery dissection and rupture [20]. Actually, improved knowledge on the relationships between facial morphometric features and other phenotypic aspects of PWS might be useful to identify which distinctive facial features are more robust and which ones are more influenced by the related clinical characteristics of the disease and its treatment, thus improving patient follow-up. For example, GH deficiency is a common condition in PWS; in addition to an overall reduction in facial growth, it might play an important role in determining craniofacial morphology. On the other hand, it has been recently demonstrated that not all facial features normalize when individuals with PWS receive recombinant human GH (rhGH) supplementation [21]. Moreover, patients with PWS are particularly susceptible to abnormalities of breathing during sleep, suffering from both central and obstructive sleep apnea. In this respect, it is well established that craniofacial alterations play a role, together with obesity, hypotonia, and hypothalamic dysfunction, in determining respiratory abnormalities [22].

The present study aimed to investigate possible associations between distinctive facial features typically occurring in patients with PWS and clinical and biochemical markers of the disease. Facial dysmorphism was evaluated through a quantitative stereophotogrammetric approach in adulthood, being the expected peculiar facial features manifested by then.

## 2. Materials and Methods

### 2.1. Subjects

Fifteen adult subjects with confirmed genetic diagnosis of PWS were recruited for the study between April 2019 and November 2021 while they were hospitalized at the Division of Auxology—Istituto Auxologico Italiano, IRCCS, Piancavallo-Verbania (Italy) for a three-week multidisciplinary body weight reduction program. Molecular genetic testing performed by Methylation-specific multiplex ligation-dependent probe amplification (MS-MLPA) or DNA polymorphism study by microsatellite analysis revealed a paternal DEL of chromosome 15q11q13 in all subjects. Their birth weight standard deviation score ranged between −3.36 and 2.28 (mean ± SD = −1 ± 1.5); their gestational week at birth was 39 ± 3.

They were aged 28 to 48 years: 8 males (42 ± 5 years) and 7 females (37 ± 8 years), chosen among white Caucasian patients with PWS who had no previous history of craniofacial injuries or surgery. All patients but one were classified as obese (BMI > 30 kg/m^2^); their BMI ranged between 23.31 and 58.63 kg/m^2^ (38.87 ± 8.92 kg/m^2^). Nine patients were treated with rhGH at the time of the study or previously during adulthood. One of these and three other patients had been treated in pediatric age; three patients had never received GH therapy. Patients received rhGH treatment for a period ranging between one and twenty years.

In addition to the patients, we assessed the facial morphometric features of 403 Caucasian healthy subjects, matched with the patients for sex and age, to serve as a reference group.

The study protocol was approved by the Ethical Committee of the Istituto Auxologico Italiano, IRCCS, Milan, Italy (reference no. 01C925, acronym: STEREOCRANPWS). In accordance with the Declaration of Helsinki, written informed consent about the purposes of the study, the experimental procedures, and the use of the collected parameters was obtained from all participants and patients’ legal representatives. 

### 2.2. Anthropometric Data and Laboratory Analyses

All PWS patients underwent physical examination, evaluation of body composition, and determination of biochemical markers of endocrine/metabolic dysfunction by standardized protocols used in the endocrinological practice. Furthermore, the nocturnal respiratory function was evaluated by oximetry test.

Height (m) and weight (kg) were used to estimate the body mass index (BMI, Kg/m^2^). Patients were considered normal-weight, overweight, or obese with BMI values < 25, between 25 and 30, or >30 Kg/m^2^, respectively; obesity was categorized as severe when BMI values were 40 Kg/m^2^ or higher [23]. Waist and hip circumferences were used to calculate the waist to hip ratio (WHR).

Body composition was measured by bioimpedance analysis (Human-IM Scan, DS-Medigroup, Milan, Italy) after 20 min of supine resting and in accordance with international guidelines [24]. Fat mass (FM) and fat free mass (FFM) were determined by using the equations specific for this population developed by Bedogni et al. [25].

Peripheral venous blood samples were drawn for the determination of insulin-like growth factor 1 (IGF-1, μg/L), glycaemia (mmol/L), insulin (mU/L), thyrotropin (TSH, mU/L), free thyroxine (fT4, ng/L), luteinizing hormone (LH, U/L), follicle stimulating hormone (FSH, U/L), cortisol (μg/dL), and adrenocorticotropic hormone (ACTH, ng/L), using laboratory standardized methods. Insulin resistance was calculated using homeostasis model assessment (HOMA-IR) [26].

Frequency of nocturnal hypoxemia episodes (arterial oxygen saturation SpO_2_ < 88%) was recorded.

### 2.3. 3D Facial Acquisition

Facial anthropometric data for both patients with PWS and healthy subjects were collected through stereophotogrammetry; participants did not experience any discomfort. Before the acquisition of the 3D facial image, an expert morphologist identified 50 facial soft-tissue anatomical landmarks by visual inspection or palpation of the face, marking the last ones on the skin with a common eyeliner. Landmarks were chosen according to international criteria [27]; a specific protocol was devised to describe the face as a whole and its different portions (facial thirds, orbital region, nose, ears, lips) and to investigate dysmorphisms associated to syndromes and genetic disorders [28,29]. The following landmarks were used in the present study:

- midline landmarks: tr, trichion; n, nasion; prn, pronasale; sn, subnasale; ls, labiale superius; sto, stomion; li, labiale inferius; pg, pogonion;
- paired landmarks: ex, exocanthion; en, endocanthion; os, orbitale superius; or, orbitale; ft, frontotemporale; zy, zygion; tr, tragion; al, alare; cph, crista philtri; ch, cheilion; go, gonion; pra, preaurale; sa, superaurale; pa, postaurale; sba, subaurale.

Subjects subsequently underwent facial 3D photograph, through a commercial stereophotogrammetric device, while sitting with a natural head position and neutral facial expression, closed mouth, and lips in a relaxed position. Healthy subjects and 13 patients with PWS were acquired at LAFAS lab, University of Milan, Italy, through a fixed system (VECTRA M3, Canfield Scientific, Fairfield, NJ, USA). The instrument consists of two cameras for each of three pods that image the face simultaneously from different points of view, thus allowing a detailed 3D reconstruction of the facial surface (capture time: 3.5 ms; geometric resolution: 1.2 mm). Due to COVID-19 restrictions, the faces of the remaining two patients were acquired at Istituto Auxologico Italiano, IRCCS, Piancavallo (VB), Italy using a portable stereophotogrammetric system (VECTRA H2, Canfield Scientific, Fairfield, NJ, USA); the instrument takes three images of the face some seconds apart, from the specific angulations suggested by the manufacturer. Three-dimensional reconstruction of all the acquired faces was obtained by VAM elaboration software (Canfield Scientific, Fairfield, NJ, USA). 

Anatomical landmarks were then manually digitized off-line on each 3D facial image and their x, y, and z coordinates were extracted, thus obtaining a geometric model of the face. From the landmark coordinates, anthropometric measurements were automatically calculated by custom computer software: 42 linear distances, 9 distance ratios, and 30 angles in all 3 spatial directions. Intra- and interobserver repeatability and accuracy of the data collection procedure were verified for both fixed and portable VECTRA devices in previous studies [30,31]. In addition, different validation studies demonstrated, through magnitude error statistics, the high comparability between facial images obtained with portable stereophotogrammetric systems and those obtained with the fixed ones, particularly when linear distances and angles are evaluated, as in the present study [31,32].

### 2.4. Statistical Analysis

All individual patients’ anthropometric variables were converted into age- and sex-specific z-scores, through the comparison with corresponding data obtained from healthy subjects (PWS patient value minus the appropriate reference group mean value, divided by the SD of the reference group value); the larger the z-score in absolute value, the more deviated the patient value from the healthy subjects one. Firstly, they were calculated separately for males and females; except for the relative position of exocanthia and nasion, respective values did not show statistically significant sex-related differences (Mann–Whitney U test, *p* > 0.05) and were pooled. Mean z-scores of patients and healthy subjects were compared using a paired Student’s t test, after checking for normal distribution of individual values. The Bonferroni correction was used to account for multiple testing (0.05/81 comparisons = *p* < 0.0006 as a threshold for statistical significance). Facial anthropometric measurements with mean z-scores at least of 1 in absolute value were considered noteworthy, since, by definition, the mean z-score of the reference group is 0 and its SD is 1. The association of each one of them with the clinical and biochemical variables was investigated by Pearson or Spearman correlation analysis, after verifying the normality of data. Given the exploratory nature of the study, and to avoid losing potential associations for unpowered analyses, the significance threshold was set at *p* < 0.05, and no correction was applied to mitigate potential false positives.

## 3. Results

Measurements concerning anthropometric characteristics, body composition, and biochemical parameters in patients with PWS are summarized in Table 1.

Row data concerning facial anthropometric characteristics for both male and female individuals with PWS are available within Supplementary Material (Table S1).

**Figure 1:**
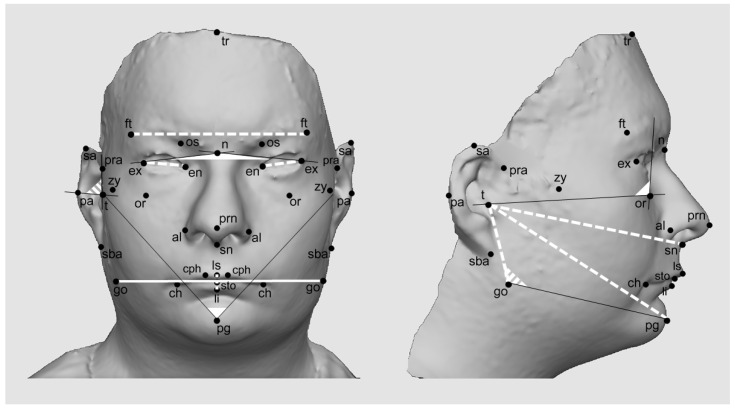
3D facial image of a subject with PWS. Black dots correspond to the anatomical landmarks used in the study; landmarks are associated with their acronyms. Anthropometric measurements with remarkable differences from healthy subjects (mean z score ≥ |1|) are highlighted. Dotted lines and angles: decreased measurements. Continuous lines and angles: increased measurements.

Analysis of facial anthropometric features revealed several significant differences when comparing patients with PWS and matched healthy subjects (Figure 1, Table 2 and Table 3). 

### 3.1. Face as a Whole

Patients with PWS showed a significantly smaller bifrontal diameter than control subjects of comparable age, ethnicity, and sex. On the contrary, a larger width of the lower face was detected. Patients with PWS showed a gradual top down decrease in facial depths, with significantly smaller middle and lower facial depths; facial convexities on the horizontal plane showed a corresponding and expected increase, with significantly larger values for lower facial convexity. Analysis of the relative position of the exocanthia and nasion, which is a morphometric measurement related to upper face convexity, detected an increase in values, with significantly greater z-scores in males than in females. Considering the facial heights on the frontal plane, only the upper portion of the face showed a slight decrease. In addition, the posterior facial height, corresponding to the mandibular ramus length, was reduced in patients. Consistently, patients did not show any significant variations in facial divergence, facial height index (FHI), and facial width/height ratio values when compared to healthy subjects.

### 3.2. Mandible

Despite the shorter mandibular ramus, the mandibular body was slightly longer in patients with PWS than in healthy subjects. Furthermore, patients showed significantly smaller gonial angles.

### 3.3. Orbital Region

Patients with PWS showed a decreased biocular width due to a significantly smaller palpebral fissure length. Furthermore, a significantly greater inclination of their orbits relative to the Frankfurt plane was detected.

### 3.4. Ears

Patients with PWS showed a smaller angle of the auricle versus the facial midplane; the left side mean z-score differed more from the reference value than the right side one. Both height and width of the ears were not different in patients and reference subjects. 

### 3.5. Lips

Patients with PWS showed a significantly smaller height of the upper vermillion, as well as a decreased lower vermillion height. In addition, the mouth height/width ratio was reduced.

No other significant difference between patients and healthy subjects was detected concerning total mouth height and width or philtrum features. 

### 3.6. Nose

None of the analyzed morphometric features of the nose showed significant differences between patients and healthy subjects.

### 3.7. Correlation Analysis

Table 4 summarizes the correlation analysis between facial anthropometric measurements and other clinical features. 

Mandible width showed a positive association with BMI, FM, glycaemia, insulin, and HOMA-IR values, while, among the other anthropometric measurements related to the mandible, the gonial angle was inversely associated with FM, glycaemia, and insulin, yet showed a positive association with the duration of GH treatment. When considering the orbital region, a relationship was observed between the inclination of the orbit and the frequency of nocturnal hypoxemia episodes. The angle of the auricle versus the facial midplane showed an inverse association with BMI values. Finally, the upper vermillion height was positively associated with insulin levels and HOMA-IR values.

Scatter plots showing the relationship between facial anthropometric measurements and biochemical/clinical parameters are available within Supplementary Material (Figure S1).

## 4. Discussion

Unlike other disorders associated with facial features typical enough to establish diagnosis, individuals with PWS show peculiar facial features, yet which are partly overlapping those of other diseases. Furthermore, they are not always evident enough to be detected through a visual inspection of the face; they manifest over time, they vary significantly among affected individuals, and they could be influenced by concurrent endocrinopathies or the related treatments. Therefore, a detailed and objective definition of facial dysmorphism associated with PWS is important both for the diagnosis and the management of the disease.

Facial phenotypic differences among genetic subtypes have been investigated in several studies, with contrasting results, differences having been detected for some features [33,34,35] or for none [36]. In the present study, a homogeneous group of patients was investigated, all of them having a paternal DEL of chromosome 15q11q13.

In nearly half of the patients participating in the study, a small birth weight for gestational age was found; this was not an unexpected finding, since a mild prenatal growth retardation with lower birth weight and length in comparison with unaffected siblings is common in PWS [1]. Nevertheless, in a recent multicentric study by Salvatoni et al. [37] on 496 newborns with PWS, lower birth weights but similar birth lengths and head circumferences were found in comparison to normative data, and a prenatal impairment in nutrition rather than in growth was suggested. The evidence of growth retardation in the physical phenotype of PWS, including the craniofacial district, is usually attributed to the relative GH deficiency associated with the syndrome [21]. Indeed, it would be interesting to investigate the possible contribution of perinatal history to the facial features in PWS.

Analysis of facial features associated with PWS showed a remarkable reduction in the bifrontal diameter when comparing patients to reference subjects, meeting the current diagnostic criteria for disease [7]. In literature, a narrow bifrontal diameter was observed in between 40% and 100% of the individuals with PWS [38]. Z-score values of the bifrontal diameter were negative in all patients examined in the current study, suggesting its usefulness for the diagnosis of the disease. Furthermore, although the correlation analysis carried out in this study should be considered exploratory due to the small sample size, no significant associations between the bifrontal diameter and PWS morbidities that could affect facial morphology emerged. This facial anthropometric feature seems to be a robust distinctive facial feature of PWS, not or scarcely influenced by coexisting endocrinopathies or their treatment.

Patients with PWS exhibited a larger lower facial width (or mandible width). The finding is in contrast with studies based on cephalometric assessments in which a smaller mandible width was reported, both in children and adult individuals with PWS [12,14,15]. Indeed, it should be noted that cephalometry evaluates skeletal characteristics of the head, while stereophotogrammetry explores the facial surface, inevitably modified by the amount of subcutaneous fat. Almost all patients examined in the present study were obese and 6 out of 15 were suffering from severe obesity. In a stereophotogrammetric study on facial features of obese subjects, Sforza et al. [39] documented an increased mandibular width. Therefore, that feature might not be a characteristic of PWS per se, but of obesity associated with it. Actually, the correlation analysis showed a significant relationship between mandible width and both BMI and FM. A positive association also emerged with glycemia, insulin levels, and insulin resistance. On the other hand, regardless of PWS, the relationship between obesity and type 2 diabetes is well known.

No significant differences were detected in facial heights when comparing PWS patients and reference subjects, except for a slight reduction in the forehead height (or upper facial height). Several cephalometric studies demonstrated smaller facial heights in PWS patients never treated with hGH [12,14,15,16]. On the other hand, studies on the effects of replacement therapy with hGH on facial growth of patients with PWS observed an increased vertical facial growth [21,40,41]. Since more than three-quarters of the patients who participated in this study were under replacement therapy with hGH or had been treated with hGH at an earlier stage of life, facial heights similar to those of healthy subjects were expected.

Patients with PWS showed smaller facial depths, more evident in its middle and lower parts, with a corresponding increase in facial convexities on the horizontal plane, in agreement with findings of the cephalometric studies already mentioned above [12,14,15,16]. Patients with PWS also showed a variation in the relative position of exocanthia and nasion with increased convexity, more marked in males, and never detected so far. Indeed, a study by de Souza et al. [21] showed that in individuals with PWS never treated with hGH, the position of the orbit changed, especially in its closest portion to the nasal root. That facial anthropometric feature might be a distinctive characteristic of PWS if it was confirmed in further studies on a larger number of affected individuals untreated with hGH therapy.

Patients with PWS showed a reduced mandibular ramus length, in accordance with cephalometric studies [12,14,15,16]; conversely, they showed a mild increase in mandibular body length, which could be due to the effect of the hGH treatment carried out by most patients. Actually, Kiellberg et al. [41] demonstrated an accelerating effect on the growth rate of the mandibular corpus length in GH-deficient boys treated with replacement therapy.

The reciprocal position of the mandibular ramus and mandibular body was also modified in patients with PWS, being the observed reduction in the gonial angle influenced by both mandibular features themselves and smaller facial depths and heights. Gonial angle was positively associated with the duration of hGH replacement therapy, gradually approaching the values of normal subjects. The angle was inversely associated with FM glycaemia and insulin levels, in accordance with the effects of GH treatment on body composition [42].

Patients with PWS showed a significantly smaller palpebral fissure length, in agreement with the quantitative data available in the literature [13,21]. This anthropometric measure could be a robust feature of PWS, as it was not associated with other clinical and biochemical markers. Within the orbital region, an increase in the inclination of the orbit versus the Frankfurt plane was also observed, never having been detected so far. This anthropometric surface measurement might be influenced by the amount of subcutaneous fat in the infraorbital region; indeed, it deserves further studies on larger and more homogeneous samples of patients, having detected an interesting association with the frequency of nocturnal hypoxemia episodes.

Auricles of patients with PWS showed no abnormalities, except for a lesser inclination versus the facial midplane, never described so far. This feature might be influenced by an increased subcutaneous fat accumulation. In fact, the inclination of the auricle was inversely associated with the BMI.

Regarding the anthropometric assessment of the mouth, patients with PWS showed a significant reduction in upper vermillion height, as expected with reference to diagnostic criteria for the disease [7]. Upper vermillion height was found to be positively associated with insulin levels and HOMA-IR. It is difficult to hypothesize the reasons for these relationships, also considering that no associations emerged with either glycemia, BMI, or FM. Interestingly, patients with PWS also showed a decreased lower vermillion height, although less evident than the upper lip, never observed before. It could be a distinctive feature of the syndrome, perhaps missed from a simple visual inspection of the face thus far, but the influence of hGH therapy cannot be excluded.

The main limitations of the study concern the size and composition of the sample. Due to the rarity of the syndrome and COVID-19 restrictions, it was possible to evaluate only a small group of patients with PWS. However, it must be considered that PWS is a rare disease, and enrolment of these patients is extremely difficult. For this reason alone, the findings of the study should be considered preliminary. On the other hand, the group of patients being small, it may be possible that the tests were underpowered and some effects not detected. We acknowledge, as another main weakness, that the patients were recruited for the study independently from hGH replacement therapy, BMI, body composition, and biochemical markers of endocrine/metabolic dysfunction. As a consequence, the non-homogeneity of the sample only allowed us to hypothesize that facial features never described so far but detected in this study through a quantitative 3D approach could be typical traits of PWS. In order to evaluate peculiar facial features, patients with PWS were compared to healthy subjects. The last ones were matched for age and sex but not for BMI, since obesity is a pathological condition, per se. The possible influence of BMI on some detected facial features was discussed; nevertheless, it should be a further limitation of the study.

The relevance of the findings is limited by the exploratory nature of the study, and subsequent dedicated studies need to be conducted for their confirmation.

The strength of our study is that all patients involved in the study were recruited and followed by a single center, with the same well-trained medical staff and the same laboratory.

## 5. Conclusions

In conclusion, this exploratory study allowed us to detail information about known distinctive features associated with PWS. Additional facial anomalies never reported in literature so far were detected; they could represent phenotypic traits of PWS, when confirmed by dedicated studies on homogeneous and larger groups of patients. Facial anthropometric features that did not show associations with clinical and biochemical markers of the disorder such as bifrontal diameter, the relative position of exocanthia and nasion, lower facial depth, mandibular ramus length, palpebral fissure length, and lower vermillion height could constitute robust distinctive facial features associated with PWS and make an important contribution to the diagnosis of the disease. Conversely, facial characteristics probably influenced by the consequences of the hypothalamic dysfunction in PWS, such as mandibular width, gonial angle, inclination of the orbit, and inclination of auricle, might have a useful role in the clinical monitoring and management of the disease.

## Figures and Tables

**Table 1:** Auxological and biochemical characteristics of the PWS study group.

WC (cm)	HIP (cm)	WHR	FM%	FFM%
112.8 ± 18.3	123.9 ± 18.9	0.92 ± 0.1	45.7 ± 8.4	53.6 ± 7.9
**Glycaemia (mmol/L)**	**Insulin (mU/L)**	**HOMA-IR**	**fT4 (ng/L)**	**TSH (mU/L)**
5.9 ± 1.8	11.2 ± 7.3	3.2 ± 2.5	11.9 ± 1.5	2.2 ± 0.7
**IGF-1 (μg/L)**	**LH (U/L)**	**FSH (U/L)**	**Cortisol (μg/dL)**	**ACTH (ng/L)**
120.6 ± 59.7	5.7 ± 7.1	18.0 ± 20.4	13.9 ± 6.8	23.4 ± 7.2

Data are expressed as mean ± SD. WC, waist circumference; HIP, hip circumference; WHR, waist to hip ratio; FM, fat mass; FFM, fat free mass; HOMA-IR, homeostasis model assessment of insulin resistance; fT4, free thyroxine; TSH, thyroid stimulating hormone; IGF-1, insulin-like growth factor-1; LH, luteinizing hormone; FSH, follicle stimulating hormone; ACTH, adrenocorticotropic hormone.

**Table 2:** 3D anthropometric measurements describing the face in toto in subjects with PWS as compared to healthy subjects.

	Anthropometric Definition	Measurement		Mean Z-score	SD	*p*
**Facial widths**	Bifrontal diameter	ft-ft		**−1.5**	0.4	0.0001 *
	Upper facial width	zy-zy		−0.9	1.1	0.0011
	Cranial base width	t-t		0.0	1.3	0.9087
	Lower facial width (mandibular width)	go-go		**1.1**	1.5	0.0045
**Facial heights**	Upper facial height	tr-n		−0.9	1.2	0.0062
	Middle facial height	n-sn		−0.2	0.8	0.4305
	Lower facial height	sn-pg		−0.6	1.2	0.0620
**Facial depths**	Upper facial depth	t_m_-n		−0.8	1.0	0.0070
	Middle facial depth	t_m_-sn		**−1.1**	0.8	0.0001 *
	Lower facial depth	t_m_-pg		**−1.3**	1.0	0.0002 *
**Facial convexities**	Relative position of the exocanthia and nasion	ex-n-ex	F	**1.1**	0.6	0.0032
			M	**2.2**	0.6	0.0003 *
	Upper facial convexity	t-n-t		0.7	1.3	0.0487
	Middle facial convexity	t-prn-t		0.7	1.1	0.0207
	Lower facial convexity	t-pg-t		**1.2**	0.9	0.0002 *
**Facial divergence**	Facial divergence	(t-n)/(pg-go)		0.0	0.7	0.9077
**Ratios**	Facial width/height	(t-t)/(n-pg)		0.5	1.1	0.1128
	Posterior/anterior facial height (FHI)	(t-go)/(sn-pg)		−0.4	0.8	0.1082

F, females; M, males; m, mid-landmark; SD, standard deviation; *p*, probability value of paired two-sided Student’s *t* test; *, *p* < 0.0006 (significance threshold after the Bonferroni correction). Mean z scores ≥ |1| are highlighted in bold.

**Table 3:** 3D anthropometric measurements describing parts of the face in subjects with PWS as compared to healthy subjects.

	Anthropometric Definition	Measurement		Mean Z-score	SD	*p*
**Mandible**	Mandibular ramus length	t_m_-go_m_		**−1.0**	0.9	0.0009
	Mandibular body length	pg-go_m_		0.8	1.0	0.0143
	Gonial angle	t-go-pg	R	**−1.6**	1.1	0.0001 *
			L	**−1.8**	1.3	0.0001 *
**Eyes**	Biocular width	ex-ex		−0.9	1.1	0.0035
	Interocular width	en-en		0.1	1.0	0.6350
	Palpebral fissure length	en-ex	R	**−1.1**	0.6	0.0001 *
			L	**−1.2**	0.9	0.0001 *
	Inclination of orbit versus the Frankfurt plane	os-or-t	R	0.8	0.9	0.0038
			L	**1.0**	0.7	0.0001 *
**Ears**	Auricle height	sa-sba	R	0.2	1.0	0.5520
			L	0.5	1.1	0.1094
	Auricle width	pra-pa	R	−0.1	1.2	0.7973
			L	0.3	1.1	0.3345
	Inclination of the auricle versus the facial midplane	ear angle	R	−0.8	1.0	0.0075
			L	**−1.2**	1.2	0.0014
**Mouth**	Mouth width	ch-ch		−0.3	1.4	0.4543
	Philtrum length	sn-ls		−0.3	1.2	0.3905
	Philtrum width	cph-cph		−0.5	0.8	0.0281
	Upper vermillion height	ls-sto		**−1.1**	0.9	0.0002 *
	Lower vermillion height	li-sto		**−1.0**	1.3	0.0049
	Mouth height/width	(ls-li)/(ch-ch)		−0.9	1.1	0.0013
**Nose**	Nasal height	n-sn		−0.2	0.8	0.4305
	Nasal width	al-al		0.7	1.0	0.0225
	Nasal protrusion	prn-sn		0.1	0.8	0.6889
	Nasal tip angle	n-prn-sn		−0.2	1.0	0.3714

R, right side; L, left side; m, mid-landmark; SD, standard deviation; *p*, probability value of paired two-sided Student’s *t* test; *, *p* < 0.0006 (significance threshold after the Bonferroni correction). Mean z scores ≥ |1| are highlighted in bold.

**Table 4:** Correlation between facial anthropometric measurements with mean z-scores of at least |1| and other clinical features in patients with PWS.

Anthropometric Measurement	BMI (kg/m^2^)	FM (%)	GH Treatment (years)	Glycaemia (mmol/L)	Insulin (mU/L)	HOMA IR	Hypoxemia Episodes (SpO_2_ < 88%)
	**Pearson r**		**Spearman ρ**				
							
Bifrontal diameter	0.18	0.25	−0.16	0.31	0.11	0.15	0.41
Relative position of exocanthia and nasion	0.12	−0.16	0.37	0.04	0.09	0.07	0.06
Middle facial depth	−0.14	−0.13	0.13	0.09	0.08	0.11	0.05
Lower facial depth	0.24	−0.06	0.17	0.39	0.21	0.28	0.28
Lower facial convexity	0.32	0.50	0.20	0.19	0.23	0.25	−0.08
Mandibular ramus length	−0.21	−0.02	−0.28	−0.21	−0.19	−0.27	−0.03
Mandibular width	**0.62** *	**0.52** *	−0.12	**0.67** **	**0.67** **	**0.74** **	0.26
Gonial angle	−0.32	**−0.62** *	**0.53** *	**−0.55** *	−0.50	**−0.56** *	−0.10
Palpebral fissure length	0.06	−0.22	0.22	0.02	0.11	0.09	−0.05
Inclination of the orbit/Frankfurt plane	−0.26	0.07	−0.16	0.01	0.05	0.10	**0.52** *
Inclination of the auricle/facial midplane	**−0.51** *	−0.24	−0.36	−0.30	0.11	−0.03	−0.27
Upper vermillion height	0.26	0.40	−0.18	0.37	**0.61** *	**0.60** *	−0.50
Lower vermillion height	0.23	0.37	−0.10	−0.04	0.18	0.15	−0.50

Pearson or Spearman exploratory correlation analysis: significant correlations are highlighted in bold (uncorrected *p*-values); ** *p* < 0.01; * *p* < 0.05. No significant correlations were found between the listed anthropometric measurements and WHR, TSH, fT4, Cortisol, ACTH, IGF-1, LH, and FSH.

## Data Availability

The data that support the findings of this study are available on request from the corresponding author. The data are not publicly available due to privacy and ethical restrictions.

## Supplementary Materials

The following supporting information can be downloaded at: https://www.mdpi.com/article/10.3390/biology11081148/s1. Table S1: 3D anthropometric measurements describing the face in toto and its parts in subjects with PWS. Figure S1: Scatter plots with linear trend line showing the relationships between facial anthropometric measurements and biochemical/clinical parameters in individuals with PWS.
