# Supplementary material for: Robustness of Distinctive Facial Features in Prader-Willi Syndrome: A Stereophotogrammetric Analysis and Association with Clinical and Biochemical Markers in Adult Individuals
Source: Biology (Basel). 2022 Jul 30;11(8):1148. doi: 10.3390/biology11081148 (PMC9405094; doi:10.3390/biology11081148)

**Figure S1**: Scatter plots with linear trend line showing the relationship between facial anthropometric measurements and biochemical /clinical parameters in individuals with PWS.

Significant associations ($p < 0.05$, uncorrected $p$ values) are depicted.

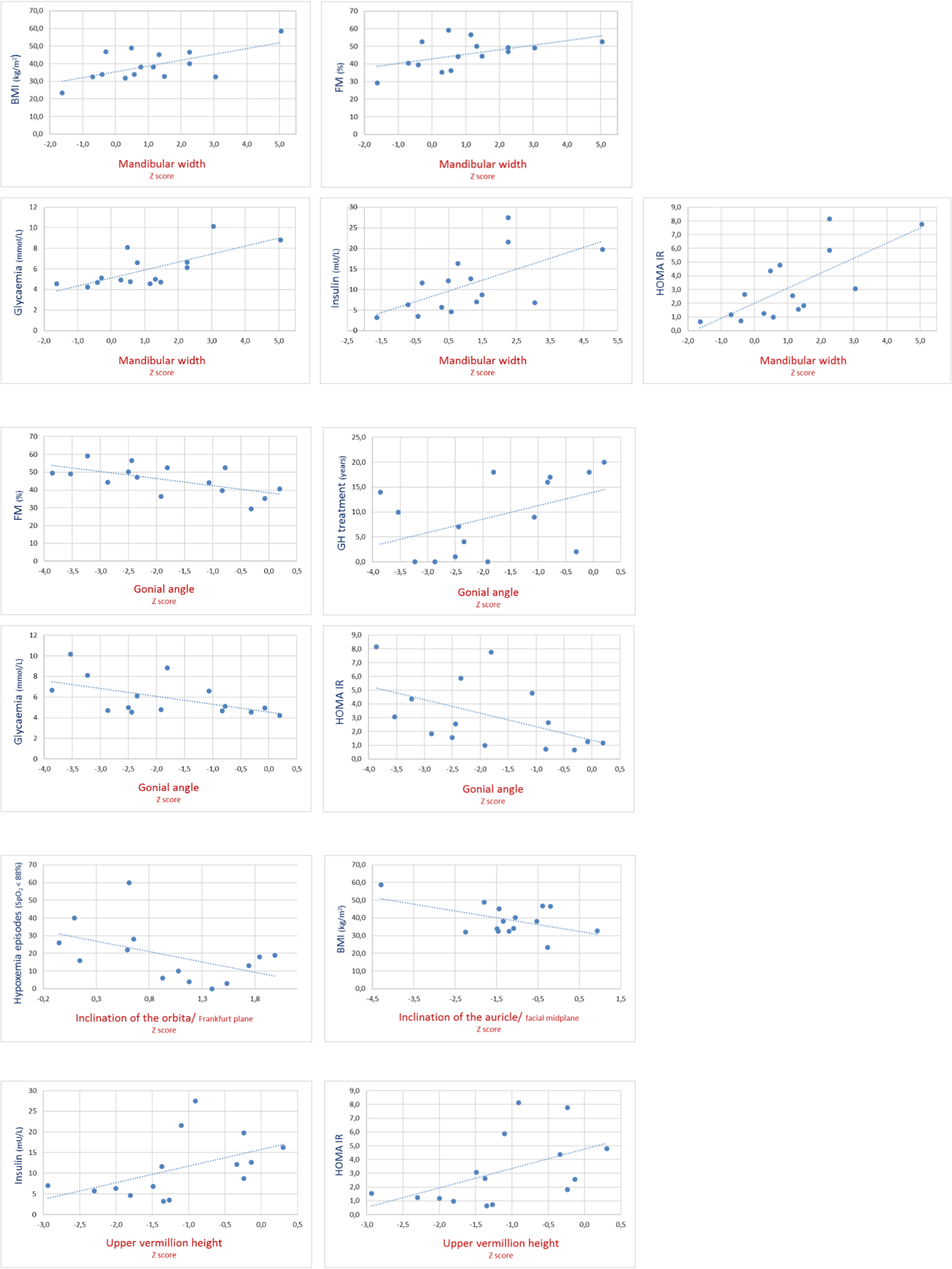

Supplement: Supplementary file 1 [file biology-11-01148-s001.zip › biology-1755462-supplementary/Figure S1.pdf]
