# Supplementary material for: Robustness of Distinctive Facial Features in Prader-Willi Syndrome: A Stereophotogrammetric Analysis and Association with Clinical and Biochemical Markers in Adult Individuals
Source: Biology (Basel). 2022 Jul 30;11(8):1148. doi: 10.3390/biology11081148 (PMC9405094; doi:10.3390/biology11081148)
Supplement: Supplementary file 1 [file biology-11-01148-s001.zip › biology-1755462-supplementary/Table S1.pdf]

Table S1: 3D anthropometric measurements describing the face in toto and its parts in subjects with PWS.

	Anthropometric definition	Measurement	Unit		Males (N = 8)		Females (N = 7)	
					**Mean**	**SD**	**Mean**	**SD**
**Facial widths**	Bifrontal diameter	ft-ft	mm		108.3	7.3	101.7	5.6
	Upper facial width	zy-zy	mm		141.7	9.1	129.5	6.7
	Cranial base width	t-t	mm		149.7	9.3	139.6	4.6
	Lower facial width (mandibular width)	go-go	mm		132.8	15.1	116.8	11.8
**Facial heights**	Upper facial height	tr-n	mm		58.4	7.9	56.0	8.3
	Middle facial height	n-sn	mm		55.4	3.9	53.8	2.8
	Lower facial height	sn-pg	mm		52.6	6.4	49.4	5.6
**Facial depths**	Upper facial depth	t_m-n	mm		97.6	4.3	91.5	4.6
	Middle facial depth	t_m-sn	mm		102.9	3.2	94.4	5.4
	Lower facial depth	t_m-pg	mm		119.6	6.8	109.0	3.9
**Facial convexities**	Relative position of the exocanthia and nasion	ex-n-ex	deg		130.3	4.6	131.0	4.8
	Upper facial convexity	t-n-t	deg		74.9	4.0	74.7	3.7
	Middle facial convexity	t-prn-t	deg		64.4	3.1	65.7	3.2
	Lower facial convexity	t-pg-t	deg		64.1	2.6	65.3	1.5
**Facial divergence**	Facial divergence	(t-n)/(pg-go)	deg		29.2	4.9	32.5	5.7
**Ratios**	Facial width/height	(t-t)/(n-pg)			141.1	14.0	137.5	8.8
	Posterior/anterior facial height (FHI)	(t_m-go_m)/(sn-pg)			117.0	15.2	110.6	13.1
**Mandible**	Mandibular ramus length	t_m-go_m	mm		61.0	6.9	54.1	4.3
	Mandibular body length	pg-go_m	mm		85.6	7.6	82.8	6.2
	Gonial angle	t-go-pg	deg	R	109.7	4.0	110.7	6.0
				L	109.3	5.1	107.5	7.2
**Eyes**	Biocular width	ex-ex	mm		90.2	6.2	83.3	4.2
	Interocular width	en-en	mm		32.4	3.3	30.0	3.3
	Palpebral fissure length	en-ex	mm	R	29.6	2.1	27.4	1.3
				L	29.5	2.8	27.2	1.3
	Inclination of orbit versus the Frankfurt plane	os-or-t	deg	R	121.6	7.3	118.3	5.4
				L	122.4	5.8	119.0	4.8
**Ears**	Auricle height	sa-sba	mm	R	65.3	4.7	58.9	3.2
				L	67.1	5.3	60.4	3.1
	Auricle width	pra-pa	mm	R	36.6	5.4	34.2	2.7
				L	38.2	3.5	34.9	4.9
	Inclination of the auricle versus the facial midplane	ear angle	deg	R	22.3	10.0	20.3	6.5
				L	21.1	10.8	19.1	4.3
**Mouth**	Mouth width	ch-ch	mm		52.7	5.1	49.3	5.2
	Philtrum length	sn-ls	mm		16.3	2.2	15.6	3.3
	Philtrum width	cph-cph	mm		11.5	2.0	10.3	1.5
	Upper vermillion height	ls-sto	mm		5.0	2.0	6.0	1.7
	Lower vermillion height	li-sto	mm		6.6	2.2	6.8	2.5
	Mouth height/width	(ls-li)/(ch-ch)			20.8	6.7	23.7	5.8
**Nose**	Nasal height	n-sn	mm		55.4	3.9	53.8	2.8
	Nasal width	al-al	mm		35.7	3.0	33.6	2.8
	Nasal protrusion	prn-sn	mm		20.9	2.4	19.9	1.4
	Nasal tip angle	n-prn-sn	deg		90.4	3.7	99.1	6.0

N, number of subjects; R, right side; L, left side; m, mid-landmark; SD, standard deviation.
